# Plasma fatty acids and primary open-angle glaucoma in the elderly: the Montrachet population-based study

**DOI:** 10.1186/s12886-021-01910-w

**Published:** 2021-03-23

**Authors:** Alicia Chemaly, Louis Arnould, Alassane Seydou, Pierre-Henry Gabrielle, Florian Baudin, Niyazi Acar, Catherine Creuzot-Garcher

**Affiliations:** 1 Department of Ophthalmology, University Hospital, 14 rue Paul Gaffarel, 21079 Dijon CEDEX, France; 2 INSERM & Dijon University Hospital, CIC1432, Clinical Epidemiology Unit, Dijon, France; 3 Centre des Sciences du Goût et de l’Alimentation, AgroSup Dijon, CNRS, INRAE, Université de Bourgogne Franche-Comté, F-21000 Dijon, France; 4 EA7460, PEC2, Cerebral and Cardiovascular epidemiology and physiopathology, Dijon, France

**Keywords:** Elderly, Glaucoma, Population-based study, Montrachet study, Fatty acids profile

## Abstract

**Background:**

To compare plasma fatty acids (FAs) between participants with primary open-angle glaucoma (POAG) and participants without neuropathy in an elderly population and to investigate specific FAs pattern in POAG.

**Methods:**

We conducted a population-based study in participants older than 75 years. Participants underwent a comprehensive eye examination with optic nerve photographs, visual field test and optic nerve OCT with RNFL thickness measurement. Glaucomatous status was defined according to the International Society for Epidemiologic and Geographical Ophthalmology classification. Lipids were extracted from plasma and FAs methylesters prepared and analyzed by gas chromatography-mass spectrometry.

**Results:**

Among the 1153 participants of the Montrachet study 810 were retained for analysis and 68 had POAG. The mean age was 82.11 ± 3.67. In multivariable analysis FAs levels were not different between POAG participants and controls (*P* = 0.078). A FAs pattern characterized by high negative weight of gamma-linoleic acid, eicosapentaenoic acid polyunsaturated FAs (PUFAs), Cis-7 hexadecenoic acid monounsaturated FAs (MUFAs) and high positive weight of eicosadienoic acid, docosatetraenoic acid, docosapentaenoic n-6, alpha linoleic acid PUFAs, eicosenoic acid MUFAs, margaric acid and behenic acid saturated FAs was positively associated with POAG. After adjustment for major confounders, individuals in the upper tertile of FAs pattern scores compared with those in the lower tertile were more likely to present POAG (OR = 3.09 [95% CI 1.29–7.40] *P* = 0.013).

**Conclusions:**

We found no significant difference regarding isolated plasma FAs between participants with POAG and participants without neuropathy in elderly but specific FAs pattern might be associated with POAG.

## Introduction

Glaucoma is the most common optic neuropathy leading to irreversible blindness worldwide [1]. It is a progressive neuropathy with multiple risk factors characterized by the destruction of retinal ganglion cells with a corresponding visual field loss. Glaucoma will affect 111.8 million people worldwide by 2040 with primary open-angle glaucoma (POAG) as the most common form [2]. Despite extensive research, its pathogenesis is still controverted. Established risk factors are age, elevated intraocular pressure (IOP), ethnic background and family history of glaucoma [3]. If elevated IOP is recognized as a major modifiable risk factor for the progression of glaucoma [4], literature shows that glaucoma can also develop under normal IOP conditions [5]. Other factors such as vascular disorder could be involved: decreased ocular blood flow could lead to ischemia and reperfusion damage of the optic nerve [6–9]. Inflammation and oxidative stress leading to microglial and complement activation are also suspected to play a role in the pathogenesis of glaucoma [10, 11].

Polyunsaturated fatty acids (PUFAs), are essential molecules of our organism. They are constituents of cell membrane, precursors of hormones such as prostaglandins and steroids and participate in the regulation of genes [12]. Linoleic acid (LA) and α-linoleic acid (ALA) are called essentials PUFAs because they cannot be synthetized and have to be ingested in the diet. They are respectively the precursors of the omega 3 and omega 6 family of PUFAs and share the same enzymatic pathway for their production [12]. An imbalance in the input or production of these PUFAs was associated with the presence of systemic [13–15] and retinal diseases [16] such as age-related maculopathy [17], diabetic retinopathy [18]. In glaucoma, PUFAs have been suspected to play a role in the pathogenesis through their inflammatory and vascular regulation activity [19]. Previous studies investigated the link between some plasma and red blood cells membrane (RBCm) fatty acids (FAs) and the presence of glaucoma, notably docosahexaenoic acid (DHA), ALA, and eicosapentaenoic acid (EPA) [19–21]. Results of these case-control studies converge towards a reduction of omega 3, especially of EPA and DHA in glaucomatous participants. Similar analysis in larger population studies are necessary to confirm these results.

The purpose of our study was to compare the total plasma FAs level between participants with POAG and participants without neuropathy in an elderly population-based study and to investigate specific FAs pattern in POAG.

## Methods

### Study design

The Montrachet (Maculopathy Optic Nerve and nuTRition neurovAsCular and HEarT diseases) population-based study was conducted from October 2009 to March 2013, in Dijon with volunteers, aged over 75 years. It was an ancillary study of the Three-City (3C) population-based study. The methodology of the Montrachet and the 3C studies has previously been described [22, 23]. All participants gave their informed consent and the study followed the tenets of the Declaration of Helsinki. The study has been approved by the Ethical Committee and registered as 2009-A00448–49. We followed the STROBE (Strengthening the Reporting of OBsevational studies in Epidemiology) statements [24] according to the EQUATOR (Enhancing the QUAlity and Transparency Of health Research) guidelines [25].

### Eye examination

Participants underwent a complete examination in the Department of Ophthalmology, in Dijon University Hospital, France. Clinical, treatment, lifestyle and demographic data as well as eating habits were collected by a self-questionnaire. The eye examination included best corrected visual acuity, slit-lamp examination, IOP measurement by air tonometry (Tonoref II, Nidek, Aichi, Japan), central corneal thickness by ultrasonic contact pachymeter (DGH 500, DGH Technology, Exton, PA, USA), axial length measurement, macular and optic disc photographs with a fundus camera (TRC NW6S, Topcon, Tokyo, Japan) after pupil dilation and a visual field for screening (Frequency-Doubling Technology, Carl Zeiss Meditec, Dublin, CA, USA). An optic nerve head spectral-domain optical coherence tomography (SD-OCT) (Spectralis, Heidelberg Engineering Co., Heidelberg, Germany) was performed, after pupil dilation with retinal nerve fiber layer (RNFL) thickness measurement around a 3.5-mm-diameter circle. The diagnosis of glaucoma was determined from photographs of optical discs interpreted by two trained ophthalmologists, blinded for clinical and RNFL SD-OCT thickness data. In case of discrepancy, a glaucoma specialist adjudicated. Persons identified as glaucomatous were further examined with gonioscopy and visual field by the Swedish Humphrey Interactive Threshold Algorithm (SITA) 24–2 (Carl Zeiss Meditec, Dublin, CA, USA). Following these examinations, they were classified into three levels of evidence according to the ISGEO (International Society for Geographical and Epidemiological Ophthalmology) classification [26]. The OCT results were therefore not taken into account (Foster et al., 2002).The 97.5 and 99.5 percentiles for the vertical cup-to-disc-ratio found in our population defined the limits of the three levels of evidence at 0.7 and 0.8 respectively [27]. In this analysis, only cases of primary open-angle glaucoma were taken into account. Severity was determined with mean deviation (MD) of Humphrey’s visual field according to the classification published by Hodapp et al. [28] An abnormal visual field test was defined when the Glaucoma Hemifield Test (GHT) was out of normal limits or at the limit [29, 30]. We included only one eye per participants and we chose the eye with the most severe POAG in case of bilateral optic neuropathy.

### Blood sampling

Blood samples were collected from fasted volunteers for plasma lipids and FAs analysis [31]. Lipids extracted from plasma were stored under inert gas then transmethylated with boron trifloride in methanol [32]. Finally, fatty acid methyl esters were isolated with hexane and analyzed by gas chromatography using a Hewlett Packard Model 5890 (Palo Alto, CA, USA) with a CPSIL-88 column (100mx0,25 mm i.d., fim thickness 0,20 μm; Varian, Les Ulis, France) equipped with a flame ionization detector. The carrier gas used was hydrogen. Sample concentration were determined by comparison to commercial and synthetic standards. The analysis focused on 25 FAs reported as a percentage of total FAs using the EZChrom Elite software (Agilent Technologies, Massy, France).

### Exclusion criteria

We excluded secondary and angle-closure glaucoma and all participants with missing data on FAs or glaucomatous status.

### Statistical analysis

Continuous variables were expressed as mean ± standard deviation (SD) or median (interquartile range) when appropriate and categorical variables as number and percentages (*n*, %). Bivariate comparisons were performed with Student test or ANOVA or Kruskal Wallis tests for continuous variables and Chi-squared or Fisher exact tests for categorical variables when appropriate. In the first step of the analysis, associations between individual plasma FAs and POAG were evaluated by a multivariable logistic regression analysis. In the second step of the analysis, in order to identify the FAs patterns (included saturated, monounsaturated and polyunsaturated FAs) associated with the presence of POAG, we used the partial least-squares (PLS) regression method. Before identifying FAs patterns, all FAs variable were centered and reduced at baseline. We retained the PLS component that was significantly associated with the presence of POAG. Identified PUFA pattern was constructed further to a score by weighting FAs concentration with factor weight values. To interpret the FA pattern, we kept those FAs with absolute values of weights ≥0.20. Individual factor scores were then categorized into tertiles. The lowest tertile of FAs pattern scores was defined as the reference group. Models were adjusted for age and sex, lipid-lowering drugs use and axial length. For all analysis, results were considered significant when *P* < 0.05. Analysis was performed using SAS software (version 9.4; SAS institute Inc.; Cary, NC, USA).

## Results

**Fig. 1:**
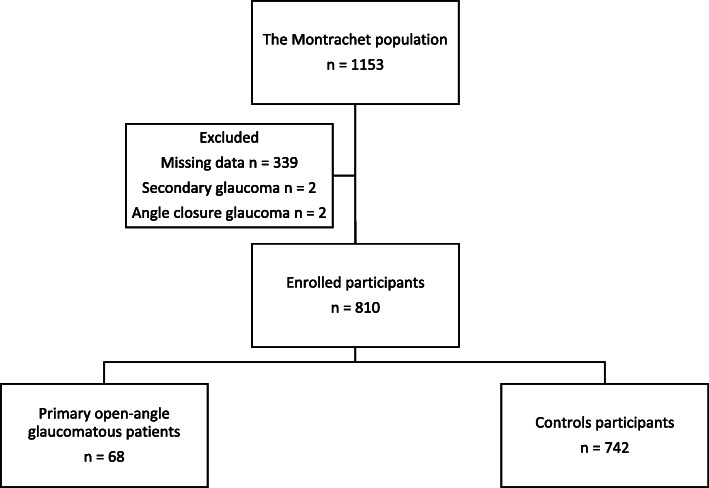
Flow chart of the study

Among the 1153 participants of the Montrachet study, 810 were included in this ancillary analysis with 68 participants with POAG (Fig. 1).

Non-participants were statistically older than participants at the inclusion, 82.59 ± 3.89 and 82.11 ± 3.67 respectively (*P* = 0.046) (Table 1). Clinical and demographic characteristics of POAG participants and participants without neuropathy are presented in Table 2. POAG participants were older (*P* < 0.003), had a higher axial length (*P* < 0.006) and higher vertical cup disc ratio (*P* < 0.001). The mean percentage of PUFAs in both groups was presented in Table 3. The univariate analysis showed that POAG participants had a significantly lower level of EPA (*P* = 0.032). There was no significant association for others FAs (including DHA) as well as for total omega 3 and omega 6. The omega 6 / omega 3 ratio was high in both groups, 7.89 ± 2.15 and 7.97 ± 1.56, respectively (*P =* 0.775).

**Table 1:** Baseline characteristics of participants to the Montrachet study (*n* = 1153), according to their inclusion in the present study

Baseline characteristics	Included	Not included ***n*** = 343	***P***
	***n*** = 810		
Age, years	82.11 ± 3.67	82.59 ± 3.89	0.046
Sex			
Male	292 (36.05)	138 (40.23)	0.179
Smoking status, yes, *n = 1132*	282 (35.43)	108 (32.14)	0.288
Alcohol consumption, yes, *n = 1017*	46 (6.30)	18 (6.27)	0.986
Body mass index	379 (46.79)	175 (51.02)	0.189
Diabetic retinopathy, *n = 1016*	7 (0.86)	2 (0.58)	1.00
Age-related macular degeneration (AMD) stage, *n = 1068*			0.350
No AMD	412 (54.71)	175 (55.56)	
Early AMD 1	235 (31.21)	102 (32.38)	
Early AMD 2	78 (10.36)	22 (6.98)	
Early AMD 3	15 (1.99)	7 (2.22)	
Late AMD	13 (1.73)	9 (2.86)	
Intraocular pressure, mmHg	15.49 ± 3.32	15.44 ± 3.64	0.844
Cup/Disc ratio *n* = 1111	0.36 ± 0.22	0.38 ± 0.21	0.179
Axial length, mm, *n = 955*	23.44 ± 1.30	23.46 ± 1.39	0.845
Antihypertensive treatment, *n = 1017*	442 (60.55)	173 (60.28)	0.937
Lipid lowering drug use, *n = 1017*	311 (42.60)	113 (39.37)	0.347
Plasma total cholesterol, mmol/l, *n = 1139*	5.80 ± 0.96	5.78 ± 0.92	0.649
Low density lipoprotein, mmol/l	3.61 ± 0.96	3.59 ± 0.81	0.759
High density lipoprotein, mmol/l	1.67 ± 0.40	1.65 ± 0.41	0.354
Triglycerides, mmol/l	1.17 ± 0.50	1.20 ± 0.55	0.384

*P* value was calculated between participants and non-participants

The results are displayed as *n* (%) for categorical variables and mean ± standard deviation for continuous variables

**Table 2:** Demographics and clinical characteristics between primary open-angle glaucomatous participants and participants without neuropathy in the Montrachet study

Baseline characteristics	Total n = 810	Participants without neuropathy ***n*** = 742	Participants with Primary open-angle glaucoma ***n*** = 68	***P***
Age, years	82.11 ± 3.67	81.99 ± 3.62	83.36 ± 4.07	0.003
Sex				
Male	292 (36.05)	266 (35.85)	26 (38.24)	0.695
Smoking status, yes, *n = 96*	282 (35.43)	257 (35.16)	25 (38.46)	0.594
Alcohol consumption, yes, *n = 730*	46 (6.30)	44 (6.58)	2 (3.28)	0.312
Body mass index, > 25 kg/m^2^	379 (46.79)	348 (46.90)	31 (45.59)	0.836
Diabetes, *n = 729*	66 (9.05)	58 (8.68)	8 (13.11)	0.248
Diabetic retinopathy	7 (0.86)	7 (0.94)	0 (0.00)	
Age-related maculopathy degeneration (AMD) stage, *n = 753*				0.530
No AMD	412 (54.71)	381 (55.14)	31 (50.00)	
Early AMD stage 1	235 (31.21)	213 (30.82)	22 (35.48)	
Early AMD stage 2	78 (10.36)	71 (10.27)	7 (11.29)	
Early AMD stage 3	15 (1.99)	15 (2.17)	0 (0.00)	
Late AMD	13 (1.73)	11 (1.59)	2 (3.23)	
Intraocular pressure, mmHg	15.49 ± 3.32	15.46 ± 3.32	15.81 ± 3.35	0.407
Cup/Disc Ratio, *n* = 780	0.36 ± 0.22	0.32 ± 0.19	0.74 ± 0.09	< 0.0001
Mean deviation of visual field, dB, *n* = 783	−11.69 ± 8.12	NA	−11.69 ± 8.12	NA
Axial length, μm, *n* = 664	23.44 ± 1.30	23.39 ± 1.24	23.88 ± 1.77	0.006
Antihypertensive treatment, *n = 730*	442 (60.55)	400 (59.79)	42 (68.85)	0.166
Lipid lowering drug use, *n = 730*	331 (42.60)	283 (42.30)	28 (45.90)	0.586
Plasma total cholesterol, mmol/l	5.80 ± 0.96	5.81 ± 0.97	5.82 ± 0.75	0.921
Low density lipoprotein, mmol/l	3.61 ± 0.84	3.60 ± 0.86	3.65 ± 0.69	0.650
High density lipoprotein, mmol/l	1.67 ± 0.40	1.67 ± 0.39	1.64 ± 0.43	0.567
Triglycerides, mmol/l	1.17 ± 0.50	1.17 ± 0.51	1.15 ± 0.43	0.781

*P* value was calculated between participants without neuropathy and participants with primary open-angle glaucoma

The results are displayed as *n* (%) for categorical variables and mean ± standard deviation for continuous variables

**Table 3:** Mean percentages of fatty acids between primary open-angle glaucomatous participants and participants without neuropathy

Chemical structure	Common name	Total	Participants without neuropathy	Participants with Primary open-angle glaucoma	***P***
Omega 6					
C18:2 n-6	Linoleic acid (LA)	25.03 ± 3.87	25.04 ± 3.89	24.95 ± 3.68	0.854
C18:3 n-6	Gamma linoleic acid (GLA)	0.45 ± 0.17	0.46 ± 0.17	0.43 ± 0.15	0.225
C20:3 n-6	Dihomo-gamma-linoleic acid (DGLA)	1.53 ± 0.32	1.53 ± 0.32	1.55 ± 0.31	0.605
C20:4 n-6	Arachidonic acid (AA)	7.46 ± 1.48	7.45 ± 1.47	7.54 ± 1.53	0.624
C22:4 n-6	Docosatetraenoic Acid (DTA)	0.26 ± 0.07	0.25 ± 0.07	0.27 ± 0.10	0.164
C22:5 n-6	Docosapentaenoic n-6 (DPA)	0.17 ± 0.05	0.17 ± 0.05	0.18 ± 0.06	0.148
Omega 3					
C18:3 n-3	Alpha linoleic acid (ALA)	0.63 ± 0.21	0.63 ± 0.21	0.68 ± 0.25	0.065
C20:5 n-3	Eicosapentaenoic acid (EPA)	1.29 ± 0.62	1.30 ± 0.64	1.13 ± 0.34	0.032
C22:5 n-3	Docosapentaenoic n-3 (DPA)	0.59 ± 0.13	0.59 ± 0.13	0.59 ± 0.14	0.957
C22:6 n-3	Docosahexaenoic acid (DHA)	2.22 ± 0.65	2.23 ± 0.66	2.15 ± 0.51	0.366
Sum					
Omega 6		35.15 ± 4.03	35.14 ± 4.08	35.22 ± 3.42	0.869
Omega 3		4.74 ± 1.30	4.76 ± 1.33	4.56 ± 0.87	0.243
Polyunsaturated fatty acids		40.36 ± 4.18	40.37 ± 4.23	40.31 ± 3.63	0.916
Ratios					
LA/ALA		43.43 ± 14.67	43.73 ± 14.92	40.12 ± 11.14	0.052
Sum Omega 6 / Omega 3		7.90 ± 2.11	7.89 ± 2.15	7.97 ± 1.56	0.775

*P* value was calculated between controls and primary open-angle glaucomatous participants

The results are expressed as mean (± standard deviation) of total fatty acids

After adjustment for age, sex, axial length and lipid-lowering drug intakes there was no significant association between EPA and POAG (*P =* 0.058) (Table 4).

**Table 4:** Associations of plasma polyunsaturated fatty acids with primary open-angle glaucoma

	Primary open-angle glaucoma	
	OR (95%, CI)	***P***
Eicosadienoic acid	2.38 (0.91–6.22)	0.078
Alpha linoleic acid	2.73 (0.77–9.65)	0.119
Eicosapentaenoic acid	0.56 (0.31–1.02)	0.058
Docosahexaenoic acid	0.82 (0.53–1.28)	0.378

After adjustments for age, sex, axial length and lipid-lowering drug use

*OR* Odd Ratio, *CI* Confidence Interval

Two hundred twelve observations were not taken into account due to missing values from axial length and lipid-lowering drug use

A plasma FAs pattern associated with the presence of POAG has been identified. This pattern explained 10.0% of the total variance in the original set of FAs. The pattern presented high negative weight of gamma-linoleic acid (GLA) and EPA PUFAs and Cis-7 hexadecenoic acid monounsaturated FAs (MUFAs). On the contrary, this pattern presented high positive weight of eicosadienoic acid, docosatetraenoic acid, docosapentaenoic n-6, ALA PUFAs, eicosenoic acid MUFAs and margaric acid and behenic acid saturated FAs (SFA) (Table 5).

**Table 5:** Weight values of 25 plasma fatty acids obtained by the partial least-squares discriminant analysis in the Montrachet study

Fatty acids		Component
Saturated fatty acids		
C12:0	Lauric acid	−0.01
C14:0	Myristic acid	−0.10
C15:0	Pentadecanoic acid	0.15
C16:0	Palmitic acid	0.07
C17:0	Margaric acid	**0.22**
C18:0	Stearic acid	0.07
C20:0	Arachidic acid	0.16
C22:0	Behenic acid	**0.28**
Monounsaturated fatty acids		
C16:1 n-7	Palmitoleic acid	−0.12
C16:1 n-9	Cis-7 hexadecenoic acid	**−0.22**
C18:1 n-7	Vaccenic acid	0.06
C18:1 n-9	Oleic acid	−0.03
C20:1 n-9	Eicosenoic acid	**0.35**
C24:1 n-9	Nervonic acid	0.08
Polyunsaturated fatty acids		
Omega 6		
C18:2 n-6	Linoleic acid (LA)	−0.04
C18:3 n-6	Gamma linoleic acid (GLA)	**−0.25**
C20:2 n-6	Eicosadienoic acid	**0.60**
C20:3 n-6	Dihomo-gamma-linoleic acid (DGLA)	0.11
C20:4 n-6	Arachidonic acid (AA)	0.10
C22:4 n-6	Docosatetraenoic acid (DTA)	**0.29**
C22:5 n-6	Docosapentaenoic acid n-6 (DPA)	**0.30**
Omega 3		
C18:3 n-3	Alpha linoleic acid (ALA)	**0.38**
C20:5 n-3	Eicosapentaenoic acid (EPA)	**−0.44**
C22:5 n-3	Docosapentaenoic acid n-3 (DPA)	0.01
C22:6 n-3	Docosahexaenoic acid (DHA)	−0.19

The fatty acids pattern obtained by Partial Least Squares Discriminant Analysis explained 10.0% of the variance in all FAs and 2.2% of the variance in response variable

Values in bold indicate interpretable FAs with absolute values of weights ≥0.20

The association of FAs pattern scores by tertiles and the presence of POAG is presented in Table 6. We found a positive and significant association between FAs pattern scores and POAG (OR _crude T3 vs. T1_ = 3.08 [95% CI 1.51–6.27], *P* < 0.01). Similar results were found after controlling for age and sex (OR _T3 vs. T1_ = 3.01 [95% CI 1.47–6.14], *P <* 0.01) and further adjustments for axial length and lipid-lowering drug intakes (OR_adj T3 vs. T1_ = 3.09 [95% CI 1.29–7.40], *P <* 0.01).

**Table 6:** Multivariable associations of fatty acids pattern scores and the presence of primary open-angle glaucoma in the Montrachet study

		Tertiles of fatty acids pattern scores		
	T1	T2	T3	
	OR (95% CI)	OR (95% CI)	OR (95% CI)	***P***
Crude	1.00 (Ref.)	2.40 (1.16–4.96)	3.08 (1.51–6.27)	0.002
M1	1.00 (Ref.)	2.46 (1.18–5.10)	3.01 (1.47–6.14)	0.003
M2	1.00 (Ref.)	2.52 (1.04–6.13)	3.09 (1.29–7.40)	0.013

*Ref* Reference, *OR* Odds ratio, *CI* Confidence interval, *T1* First tertile, *T2* Second tertile, T3 = third tertile

M1: age and sex-adjusted model

M2: M1 with further adjustment for axial length and lipid-lowering drug use

Two hundred twelve observations were deleted due to missing values from axial length and lipid-lowering drug use

## Discussion

In this population-based study we compared plasma FAs levels in participants with POAG and in participants without neuropathy by means of two methods: FAs individually and covariance analysis. We did not find any difference regarding plasma FAs level in POAG participants compared to participants without neuropathy after multivariable analysis.

Our results are not in line with previous studies which found an association between the level of plasma and RBCm FAs and glaucoma [19–21, 33]. Ren et al. reported a decrease of EPA, DHA, ALA, and total omega 3 in glaucoma with a linear association with severity of POAG [20]. Yu et al. found a decrease of EPA only in severe NTG [21]. Acar et al. found that DHA level in RBCm was decreased in POAG patients in the pre-clinical stage [19]. At the opposite, Yuki did not find any association between NTG and FAs [33]. Contrary to our study, these studies were conducted with univariate statistical analysis and they included a smaller selected population. Moreover our population based study analyzed older age participants with a mean age of 82 years.

We found a high ratio of omega 6 / omega 3 in the two groups. According to the nutritional recommendations this ratio should be comprised between 2 and 3 in order to reduce the risk of cardiovascular and neurodegenerative diseases [17]. Moreover, according to the nutritional AFSSA recommendation the linoleic acid (LA) / ALA ratio should be around 5 [34]. In this study both groups are 6 times superior to this recommendation [34]. This is in line with our previous findings showing that Montrachet population was in very good health.

We identified a specific plasma FAs pattern significantly associated with POAG. After adjustment for major confounders, individuals in the upper tertile of FAs pattern scores compared with those in the lower tertile were more likely to present POAG. This FAs parttern seems to be associated with POAG in our old population. The analysis of a FAs pattern might be more appropriate to investigate the pathogenesis of glaucoma rather than considering FAs individually.

The involvement of FAs in the pathogenesis of glaucoma could intervene at three different levels. First, FAs have an anti-inflammatory and neuroprotective effect in the retina by preventing the apoptosis of retinal cells and by regulating glia [16]. They are also major components of cell membranes and therefore have a key role in the fluidity and the aggregability of RBC [35, 36]. An imbalance in their composition could lead to decreased ocular blood flow and optic nerve perfusion pressure as it is found in glaucomatous patients [37]. Finally, they are known to be the precursors of prostaglandins, well known for these hypotonizing and anti-inflammatory effects.

In that perspective, previous studies have investigated if diet modification could have a protective effect. Nguyen demonstrated in rats that rich diet of omega 3 FAs causes better retinal ganglion cell function, and similarly decreases IOP. Similarly, deficiency in omega 3 FAs causes dysfunction [38–40]. In humans, no strong association have been established between PUFAs intakes and glaucoma. Renard suggested in a recent observational study on a French glaucomatous population that omega 3 fatty acids intake have a protective effect against POAG [41]. A recent observational population-based study suggest that an increased DHA and EPA and global omega 3 intake decrease the risk of glaucoma only if global PUFAs level intake is controlled [42]. A randomized controlled trial conducted by Garcia Medina did not find any improvement in glaucoma with anti-oxidants (with and without omega 3) intake [43]. Moreover, Kang found that a globally high omega 3 / omega 6 ratio increases the risk of glaucoma [44].

As no strong evidence of specifics FAs implication was demonstrated in our study, the benefit of supplementation in some FAs remains debated.

We acknowledge several limitations to this study. First, participants of Montrachet study are Caucasian, voluntary, elderly and from an urban population. These volunteers follow a healthy lifestyle. We could not extrapolate our results to another group. Second, we included uneven number of participants in the two groups because there were not many subjects diagnosed with glaucoma and therefore limits the interpretation of the results. Furthermore, the low number of participants with primary open-angle glaucoma could have reduced the statistical power of the analysis. Third, our study did not include any analysis of the food intake of the participants. Fourth, we used only plasma analysis of FAs. This could lead to bias of measure, as it only reflects recent dietary intake compared to RBCm FAs analysis [45, 46].

In conclusion, there is no significant difference regarding plasma FAs level in multivariate analysis when FAs are analyzed isolated. The global study of FAs highlighted a specific FAs pattern associated with POAG. The clinical relevance of this pattern warrants further studies.

## Data Availability

The data that support the findings of this study are available from the 3C Committee but restrictions apply to the availability of these data, which were used under license for the current study, and so are not publicly available. Data are however available from the authors upon reasonable request and with permission of 3C Committee (E3C.U708@inserm.fr or louis.arnould@chu-dijon.fr).
